# Advancing clinical management of left ventricular thrombosis: prevention, detection and treatment modalities in the modern era

**DOI:** 10.1136/heartjnl-2024-324605

**Published:** 2025-02-12

**Authors:** Qian Zhang, Haikuo Zheng, Zhongfan Zhang, Yuanzhen Xu, Wenqi Zhang

**Affiliations:** 1 China-Japan Union Hospital of Jilin University, Changchun, Jilin, China

**Keywords:** Cardiovascular Diseases

## Abstract

Heightened interest in left ventricular thrombus (LVT) stems from the consistent association of subsequent stroke and systemic embolism after LVT, and many aspects of its management still exist in a grey area of evidence. The current delay in intervention is likely related to a limited understanding of the disease pathophysiology, along with an underestimation of LVT by standard imaging modalities. With the rapid development of antithrombotic regimens consisting of direct oral anticoagulants (OACs), which have shown early safety and efficacy, there is a growing need to understand and accurately diagnose the LVT process in order to determine appropriate management solutions. This educational review will oversee LVT pathophysiology, current status of the guidelines-recommended echocardiographic approach and the role of multimodality imaging, as well as prevention and treatment modalities in the modern era. Meanwhile, the review proposes an algorithm for the prevention and treatment of LVT based on current guidelines and expert consensus, and highlights the need for more investigations to identify risk stratification methods for individual patients, and lastly, discusses the potential of direct OACs in the management of LVT.

## Introduction

 Although the management of cardiovascular diseases has markedly improved over the last decades, left ventricular thrombus (LVT) formation after non-ischaemic cardiomyopathies (NICM) and ischaemic cardiomyopathies (ICM) still remains a challenging complication given the high risk for subsequent stroke and systemic embolism.^1 2^ There are only limited guideline recommendations and expert consensus documents with regard to decisions concerning the diagnosis, prevention and treatment of LVT.^3^,^6^ Further complicating the matter, most evidence that forms the basis of current guidelines and expert consensus documents is from an earlier era with markedly different management practices. Indeed, some emerging evidence favours an antithrombotic regimen consisting of direct oral anticoagulants (OACs), but whether this can be extrapolated to all patient populations with LVT has not been thoroughly investigated in randomised clinical trials (RCTs).^7^,^10^ Meanwhile in the modern era of emphasising early imaging detection to decrease potentially fatal complications, diagnostic imaging modalities still vary between centres.^11^ In this educational review, we overview the pathogenesis, diagnosis and current management of LVT, and aim to provide a comprehensive view of the available evidence on LVT to help clinicians make better decisions when dealing with patients with LVT.

### Pathophysiology

**Figure 1:**
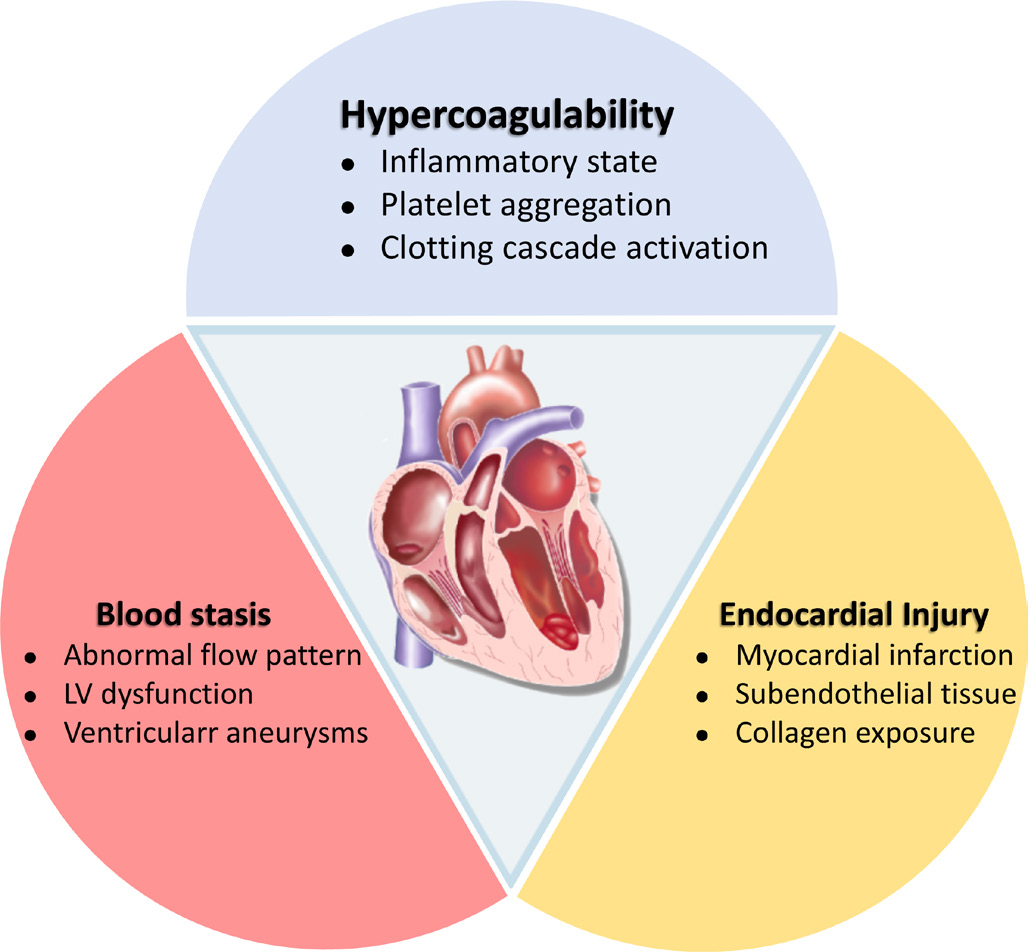
Virchow’s Triad: pathophysiological basis. LV, left ventricular.

Most cases of LVT were associated with ICM, especially after anterior myocardial infarction, while the rest were attributed to NICM, such as dilated cardiomyopathy (DCM), Takotsubo syndrome, left ventricular (LV) myocardial densification insufficiency, eosinophilic myocarditis, perinatal cardiomyopathy, and cardiac amyloidosis.^12^ The Virchow’s triad, consisting of ventricular wall tissue damage, blood stasis and an inflammatory/hypercoagulable state, represents the primary mechanism for LVT formation (figure 1).^13 14^ In the settings of ICM, the risk of LVT occurrence is determined by the onset of ischaemic symptoms and the timing and duration of the intervention.^15 16^ The intensity of myocardial injury is strongly associated with susceptibility to LVT, especially after acute ischaemic events. Blood stasis is primarily attributable to impaired LV function, characterised by reduced LV ejection fraction (LVEF) and diminished or dysfunctional large-scale motion in the LV apex or anterior wall.^17 18^ These factors collectively contribute to the formation of abnormal blood flow vortexes, ultimately resulting in blood stasis. Additionally, the stagnation of blood flow resulting from overall LV dysfunction may also represent a critical factor in the intraventricular thrombosis of NICM.^13^

The inflammatory response triggered in the local tissue injury, the low shear stress environment in the infarction area, coupled with reduced LVEF, collectively activate the coagulation cascade. This activation leads to fibrin cross-linking and platelet and red blood cell aggregation, ultimately culminating in the formation of fresh thrombus.^19^ A comprehensive understanding of tissue damage, blood stasis and fibrin cross-linking in the thrombotic process provides a theoretical foundation for prioritising anticoagulation therapy over sole antiplatelet therapy in patients with LVT. Furthermore, some studies also emphasise the key role of proinflammatory and hypercoagulable states in the development of LVT.^14^ Indeed, the persistent hypercoagulable state following acute myocardial infarction (AMI) can extend for up to 6 months. Elevated levels of inflammatory markers (eg, C reactive protein, fibrinogen) and increased neutrophil-to-lymphocyte ratios in serum on admission have been established as independent predictors of early LVT occurrence following AMI.^20 21^ Concurrently, the involvement of inflammatory responses, the hypercoagulable nature of blood, and specific disease processes affecting endocardial health (such as amyloidosis and eosinophilic myocarditis), represent significant pathophysiological components of NICM and substantially increase the risk of LVT.^22 23^

### Diagnostic modalities

#### Transthoracic echocardiography

**Figure 2:**
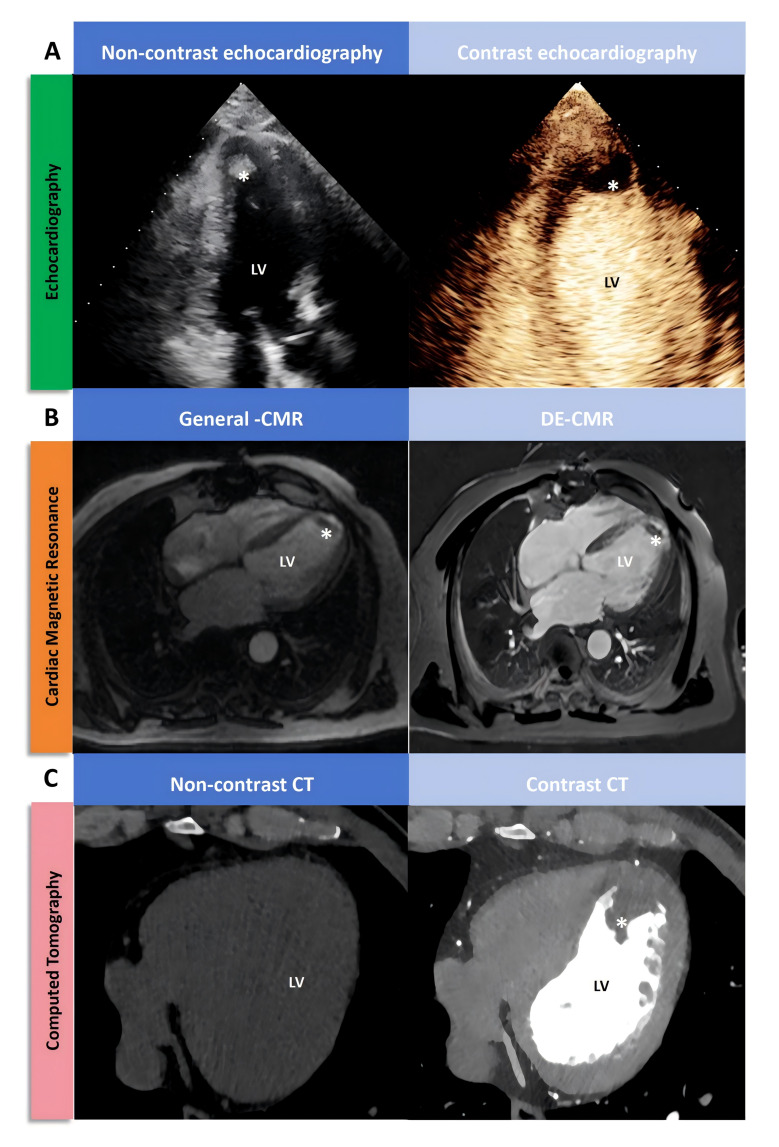
Visual representation of left ventricular thrombosis. CMR, cardiovascular MR; DE-CMR, delayed enhancement cardiovascular MR; LV, left ventricular.

Transthoracic echocardiography (TTE) remains the primary guideline-recommended imaging technique for assessing the presence, shape and size of LVT. However, the major inherent limitation of TTE in detecting LVT is inaccurate localisation of the LV apex due to abnormal chest anatomy and poor visualisation of the echocardiographic window. TTE demonstrates high specificity and sensitivity in detecting LVT when optimal visualisation of cardiac structures is attainable. TTE criteria for LVT diagnosis include: (1) Well-defined, echogenic intracavitary mass distinct from myocardial tissue, (2) Clear demarcation from surrounding ventricular cavity, (3) Complete separation from endocardium and (4) Persistence throughout the cardiac cycle. Careful differentiation from pseudotendons and trabeculae is essential. Moreover, artefacts (reverberation, lateral flaps, near-field artefacts) can mimic thrombus and should be meticulously excluded to avoid diagnostic errors (figure 2A).^24^ Additionally, patients with suboptimal chest anatomy, including narrow intercostal spaces, obesity, thoracic deformities, or pulmonary conditions such as emphysema, can pose challenges for TTE in visualising the LV apex and detecting LVT. This limitation may result in diagnostic uncertainty in 10%–46% of LVT cases, compromising sensitivity.^25^ TTE with contrast can improve endocardial border definition, thereby increasing sensitivity to approximately 64% while preserving specificity comparable to conventional TTE.^26^ Nevertheless, small or non-protruding LVT may remain undetected even with TTE with contrast.^24^

#### Cardiovascular MR

Comparative studies consistently demonstrate the superiority of cardiovascular MR (CMR) over TTE in detecting LVT. Delayed enhancement CMR (DE-CMR) in particular,^27 28^ often considered the gold standard despite lacking surgical/pathological validation, excels in characterising thrombus presence, size and location. While Cine-CMR without contrast has been shown to miss 44%–50% of LVT cases, the sensitivity of DE-CMR reaches 88% by leveraging tissue characterisation rather than solely relying on anatomical features.^27^ However, the high cost, time-consuming nature and limited accessibility of CMR restrict its widespread application in imaging diagnosis. Moreover, differentiating no-reflow zones from LVT using CMR remains challenging, necessitating careful interpretation and further histopathological studies to refine the thrombus characterisation capabilities of DE-CMR (figure 2B).

#### Computed tomography

CT demonstrates sensitivity and specificity comparable to TTE in LVT detection (figure 2C). However, accurate LVT visualisation necessitates contrast enhancement, as non-contrast CT is insufficient.^29^ Contrast-enhanced multidetector CT typically identifies LVT as an LV filling defect. While comparing arterial and delayed phases offers limited tissue characterisation, spectral CT material decomposition techniques (based on iodine and blood) show potential for improved thrombus assessment. Nevertheless, both contrast-enhanced multidetector CT and spectral CT involve intravenous contrast administration and radiation exposure.^30^ Moreover, their sensitivity is inferior to CMR, limiting their routine clinical application. Hence cardiac CT angiography is commonly considered a valuable alternative diagnostic modality.

#### Radioactive labelling

In the 1980s, radioactive labelling of blood components within mural thrombus emerged as a potential LVT diagnostic modality.^31 32^ Indium-111 labelled platelets demonstrated superior imaging performance compared with TTE, achieving 100% specificity and 71% sensitivity in LVT detection.^32^ However, the time-consuming nature and high cost of this method, and radiation exposure due to this method limited its widespread adoption. Additionally, it is ineffective for small thrombi, requiring active platelet aggregation on the thrombus surface for optimal performance.^32^ Diagnostic challenges arise in patients with elevated left hemidiaphragms due to potential spleen-apex activity confusion and in those with large LV aneurysms where stagnant blood can mimic thrombus formation, potentially leading to misdiagnosis. Thus this diagnostic modality is currently not widely used in clinical practice.

**Figure 3:**
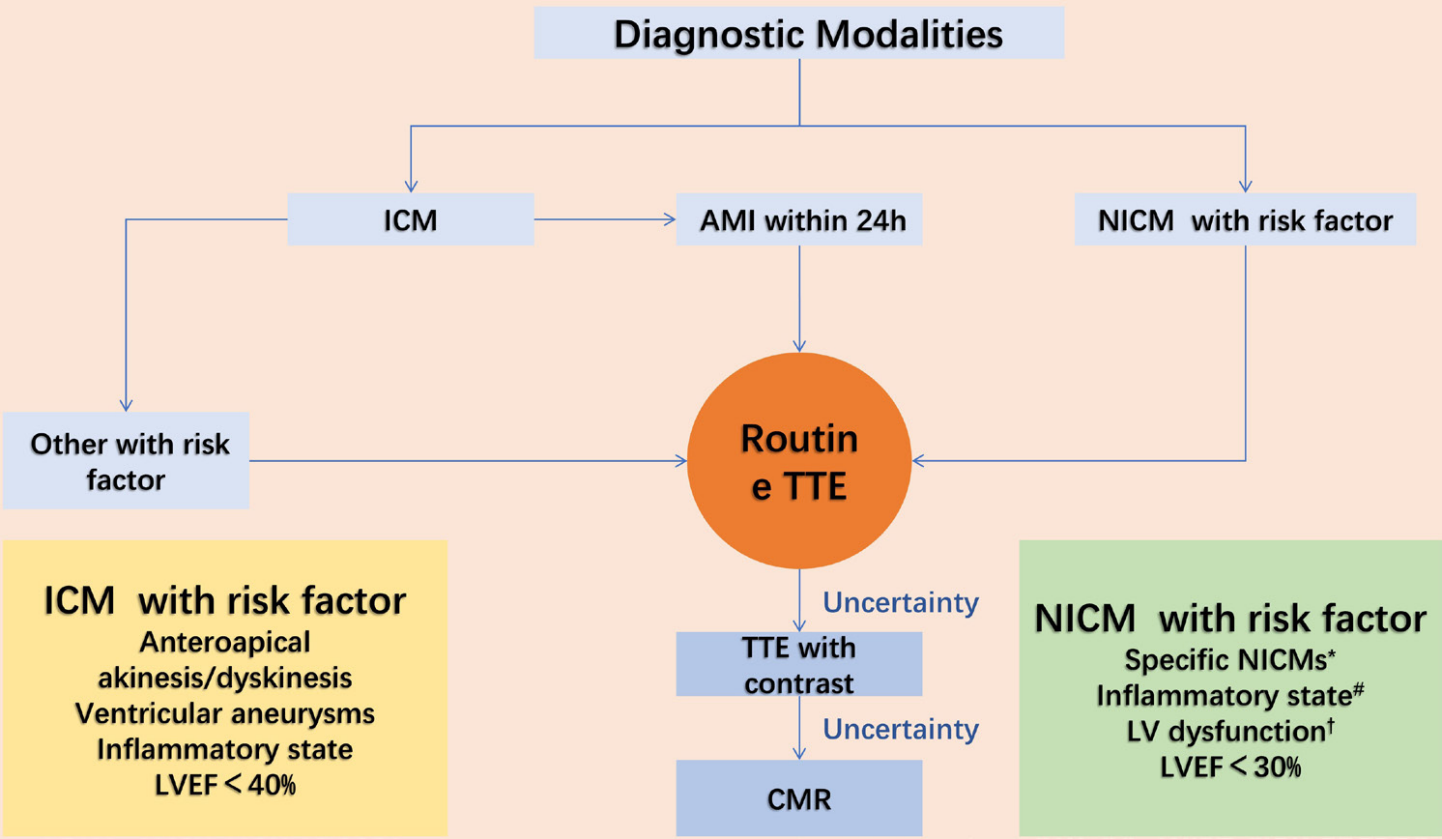
Proposed diagnostic modalities for left ventricular thrombosis. AMI, Acute myocardial infarction; CMR, cardiovascular MR; ICM, ischaemic cardiomyopathies; LV, left ventricular; LVEF, left ventricular ejection fraction; NICM, non-ischaemic cardiomyopathies; TTE, transthoracic echocardiography. *Dilated cardiomyopathy, Takotsubo syndrome, left ventricular myocardial densification insufficiency, eosinophilic myocarditis, perinatal cardiomyopathy, or cardiac amyloidosis. #C reactive protein, fibrinogen and the neutrophil-lymphocyte ratio. †Reduced ejection fraction and/or large apical or anterior LV akinesis or dyskinesis (ie, aneurysm) and reduced contractile function facilitating stasis caused by an abnormal vortex.

To optimise the early diagnosis and management for LVT, we propose an algorithm for diagnostic modalities: (1) Routine TTE is performed as the primary diagnostic tool, and contrast TTE is supplemented in case of diagnostic difficulties; (2) If the diagnosis is still not confirmed, use CMR; (3) CT was chosen when CMR was unavailable or not tolerated. In patients with AMI, imaging is performed within 24 hours of the onset of symptoms (figure 3).

### Prevention and treatment of LVT

#### Prevention

Prevention focuses on NICM with depressed LV systolic function and AMI. Most of the preventive data on ICM and NICM of LVT originate from retrospective studies. Not enough RCTs have prospectively evaluated OACs for this indication. Consequently, current preventive strategies for high-risk individuals rely heavily on limited data from related fields to inform treatment decisions. In the absence of robust evidence, guideline-directed medical prevention for LVT is currently limited to ST segment elevation myocardial infarction (STEMI) involving anteroapical akinesis/dyskinesis, with vitamin K antagonists (VKA)-related antithrombotic regimens as the current first-line therapy. Specific recommendations on NICM are rare. This educational review summarises the current guidelines and expert consensus documents regarding LVT prevention, as shown in table 1.

**Table 1:** Guidelines/consensus on the prevention of LVT

Guidelines	Recommendation	INR range	Duration	Class of recommendation	Level of evidence
2012 ACCP Guidelines^51^	In patients with high risk of LVT following anterior myocardial infarction, VKA can be added to DAPT after drug-eluting stent implantation.	2.0–3.0	3–6 months	II	C
2013 ACC/AHA STEMI Guidelines^42^	In patients with STEMI who exhibit anterior apical akinesis or dyskinesis, anticoagulation with VKA may be considered.	During combined DAPT (2.0–2.5)	3 months	IIb	C
2014 ASA/AHA Stroke Prevention Guidelines^41^	VKA anticoagulation may be considered in patients with ischaemic stroke or TIA who have experienced STEMI and exhibit anterior apical akinesis or dyskinesis.	Around 2.5(2.0–3.0)	3 months	IIb	C
2016 AHA scientific statement on DCMs^52^	Anticoagulation is reasonable in patients with peripartum cardiomyopathy and severe LV dysfunction to prevent thrombus formation given the risk of hypercoagulable state during pregnancy.	–	–	–	C
2016 The ESC Heart Failure Association^53^	Heparin-based anticoagulation in acute peripartum cardiomyopathy with LVEF≤35% to decrease the risk of thromboembolism.	–	–	–	–
2017 CCS HF Guidelines^54^	Recommend against routine anticoagulation after large anterior MI and low EF, in the absence of intracardiac thrombus or other indications for anticoagulation.	–	–	–	–
2018 International Expert Consensus on Takotsubo Syndrome^5^	Severe LV dysfunction with extended apical ballooning entails the risk of an LV thrombus and subsequent systemic embolism anticoagulation with intravenous/subcutaneous heparin would appear to be appropriate in such patients and postdischarge.	–	–	–	–
2021 AHA/ASA Stroke Prevention Guidelines^55^	For patients with acute anterior myocardial infarction and reduced EF but no evidence of LVT, empirical anticoagulation may be considered.	–	At least 3 months	2b	C
2022 Scientific StatementFrom the AHA^3^	OAC could be considered in patients with specific types of DCM at increased risk for LV thrombus formation such as those with Takotsubo syndrome, LV non-compaction, eosinophilic myocarditis, peripartum cardiomyopathy and cardiac amyloidosis. OAC is implemented in such cases.	–	–	–	–

ACC, American College of Cardiology; ACCP, American College of Chest Physicians Guideline; AHA, American Heart Association; ASA, American Stroke Association; CCS, Canadian Cardiovascular Society; DAPT, dual antiplatelet therapy; DCM, dilated cardiomyopathy; EF, ejection fraction; ESC, European Society of Cardiology; HF, heart failure; INR, international normalised ratio; LV, left ventricular; LVEF, left ventricular ejection fraction; LVT, left ventricular thrombus; MI, myocardial infarction; OAC, oral anticoagulant; STEMI, ST segment elevation myocardial infarction; TIA, transient ischaemic attack; VKA, vitamin K antagonist.

In terms of AMI, RCTs from the prethrombolytic and thrombolytic eras (1970s–1990s) provided robust evidence supporting anticoagulation strategies for LVT prevention.^33^ Meanwhile, a meta-analysis of 11 studies (856 patients) revealed a 5.5-fold increased risk of thromboembolic events in patients with acute anterior myocardial infarction with LVT compared with those without LVT.^34^ Anticoagulation reduced LVT formation by 68% and decreased systemic embolism risk in these patients. However, these guideline-supporting studies predate the modern reperfusion era, characterised by limited dual antiplatelet therapy (DAPT) use and under-reported postanticoagulation complications. Therefore, carefully evaluating the balance between the benefits and adverse prognostic risks of prophylactic anticoagulation in patients with AMI receiving DAPT is crucial in contemporary practice. Driven by extensive clinical investigations on OAC, recent advancements in antithrombotic strategies have emerged. Concurrently, growing recognition of the heightened bleeding risk associated with VKA therapy challenges the prophylactic use of VKAs in high-risk patients. Le May *et al* retrospectively analysed 460 patients with postpercutaneous coronary intervention with anterior STEMI and reduced/absent apical motion, comparing outcomes between those receiving and not receiving VKA therapy. Among patients receiving DAPT as baseline antiplatelet therapy for anterior STEMI, the VKA group experienced a significantly higher risk of net adverse clinical events (mortality, stroke, re-infarction, major bleeding) within 180 days compared with the non-VKA group.^35^ Shavadia *et al* similarly found no reduction in ischaemic events but increased bleeding rates with prophylactic VKA in 398 patients with anterior STEMI and LV dysfunction. However, the observational nature of these studies predisposes them to significant bias.^36^ Collectively, historical clinical trials indicate that short-term prophylactic anticoagulation may reduce the risk of LVT in patients with anterior myocardial infarction. However, these trials are underpowered to determine whether this prophylactic anticoagulation leads to a clinically significant reduction in systemic embolism or major adverse cardiovascular events (MACE).

In terms of NICM with depressed LV systolic function, no RCTs support the routine use of OAC for the primary prevention of LVT in these patients. Considering the variety of NICM, current consensus documents also do not enable systematic recommendations for NICM-related LVT prevention. However, there are also documents emphasising that OAC could be considered in patients with specific NICM, such as those with Takotsubo syndrome, LV myocardial densification insufficiency, eosinophilic myocarditis, perinatal cardiomyopathy and cardiac amyloidosis.^337^,^39^ Notably, the recommended duration for prophylactic OAC in these NICM subtypes remains undetermined when OAC is implemented. The preventive duration requires individualised adjustment by clinicians based on the patient’s condition, undeniably complicating clinical management.

**Figure 4:**
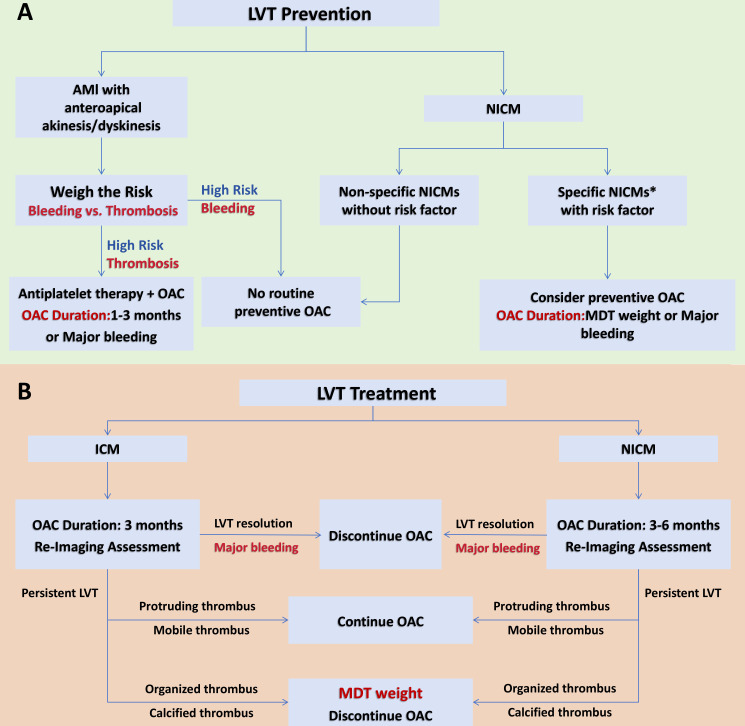
Proposed prevention modalities for LVT. AMI, acute myocardial infarction; ICM, ischaemic cardiomyopathies; LVT, left ventricular thrombus; MDT, multidisciplinary team; NICM, non-ischaemic cardiomyopathies; OAC, oral anticoagulants. *CT was chosen when cardiovascular MR was unavailable or not tolerated.

Based on current guidelines and expert consensus documents, we propose an algorithm for LVT prevention (figure 4A). For patients with AMI involving anteroapical akinesis/dyskinesis, prophylactic anticoagulation may be initiated for 1–3 months, if the risk of thrombosis is high. For patients with specific types of NICM, the decision to initiate prophylactic anticoagulation may be determined by a multidisciplinary assessment weighing bleeding risks and benefits, with OAC duration determined by the multidisciplinary team (MDT) on a case-by-case basis.

### Treatment

Indium-111 platelet imaging studies consistently reveal that most LVT, irrespective of age or size, exhibit continuous platelet accumulation on their exterior, which highlights the persistent platelet activity on the thrombus surface and the associated risk of embolism.^32 40^ Most current international guidelines suggest VKAs as first-line therapy.^41 42^ However, only limited evidence suggests anticoagulation therapy can effectively mitigate the embolism complications associated with LVT, with the fibrinolytic mechanism inherent in anticoagulants playing a pivotal role in promoting LVT dissolution. The optimal duration of anticoagulation for the treatment of LVT is uncertain, with no RCTs investigating alternative durations of therapy to date. Results from the LEVITATION Survey show that most centres are opting for an anticoagulation duration of 3–6 months.^11^ This educational review summarises the current guidelines and expert consensus documents regarding LVT treatment, as shown in table 2.

**Table 2:** Guidelines on the treatment of LVT

Guidelines	Recommendation	INR range	Duration	Class of recommendation	Level of evidence
2012 ACCP Guidelines^51^	Patients with anterior myocardial infarction and confirmed LVT undergoing drug-eluting stent implantation should be treated with VKA anticoagulation in conjunction with DAPT.	2.0–3.0	3–6 months	II	C
Ninth ed: American College of Chest Physicians Guidelines^56^	VKA therapy is probably indicated for patients with image-proven intracardiac thrombus in the setting of cardiac amyloidosis.	–	–	–	–
2013 ACC/AHA STEMI Guidelines^42^	VKA may be considered in patients with STEMI and asymptomatic LVT.	During combined DAPT (2.0–2.5)	3 months	IIa	C
2014 ASA/AHA Stroke Prevention Guidelines^41^	For patients with ischaemic stroke/TIA and acute myocardial infarction complicated by LVT, VKA anticoagulation is generally recommended. If VKA therapy is not tolerated, low-molecular-weight heparin or NOACs can be considered as alternative options.	Around 2.5(2.0–3.0)	3 months	I	C
2015 STEMI Guidelines of China^57^	Warfarin anticoagulation is a reasonable option for patients with STEMI and asymptomatic LVT.	During combined DAPT (2.0–2.5)	3–6 months	IIa	C
2016 scientific statement from AHA for specific DCM^52^	Patients with DCM need at least 3 months of OAC therapy for LV thrombus	–	–	II	C
2017 ESC STEMI Guidelines^4^	Oral anticoagulation may be considered for patients with LVT. Echocardiography should be repeated to guide treatment decisions and assess the risk of bleeding, as well as the potential impact of antiplatelet factors.	–	6 months	IIa	C
2021 AHA/ASA Stroke Prevention Guidelines^55^	For patients with stroke or TIA and LVT, anticoagulation with VKA is recommended to reduce the risk of recurrent stroke.	–	At least 3 months	I	B
2022 Scientific Statement From the AHA^3^	NICM with LV thrombus should be treated with OAC for at least 3–6 months, with discontinuation if LVEF improves to >35% (assuming resolution of the LV thrombus) or if major bleeding occurs.	–	–	–	–
2023 ESC ACS Guidelines^58^	Oral anticoagulant therapy (VKA or NOAC) should be considered in patients with confirmed LVT.	–	3–6 months	IIa	C

ACC, American College of Cardiology; ACCP, American College of Chest Physicians Guideline; ACS, acute coronary syndrome; AHA, American Heart Association; ASA, American Stroke Association; DAPT, dual antiplatelet therapy; DCM, dilated cardiomyopathy; ESC, European Society of Cardiology; INR, international normalised ratio; LV, left ventricular; LVEF, left ventricular ejection fraction; LVT, left ventricular thrombus; NICM, non-ischaemic cardiomyopathies; NOAC, new oral anticoagulant; OAC, oral anticoagulant; STEMI, ST segment elevation myocardial infarction; TIA, transient ischaemic attack; VKA, vitamin K antagonist.

But the present dilemma is the persistence of a proportion of thrombus after 3–6 months of anticoagulation, and evidence on how to deal with persistent LVT is lacking for this particular subgroup. Protruding and mobile thrombus is perceived to be more likely to embolise than immobile, calcified and organised thrombus.^43 44^ Hence, prolonged anticoagulation and repeated imaging assessment are generally recommended in protruding and mobile thrombus until LVT total regression; on the other hand, in patients with immobile, calcified and organised thrombus, the risk of embolisation may be low, and discontinuation of anticoagulation is probably a reasonable option after a MDT risk-benefit discussion. However, due to the lack of high-quality evidence, decisions regarding the modality and duration of anticoagulation should be made on a case-by-case basis. Additionally, there are insufficient data that surgical excision for persistent LVT has net efficacy, which is performed only in extreme clinical cases (eg, intolerance of anticoagulation with high risk of cardiogenic embolisation).^12^

Lastly, based on current relevant guidelines and expert consensus documents, we propose an algorithm for LVT treatment, as shown in figure 4B.

### New OACs

Given their established efficacy in treating atrial fibrillation (AF) and venous thromboembolism, it is reasonable to explore the potential benefits of new OACs (NOACs) in the management of LVT. While several guidelines recommend NOACs as a promising alternative to VKAs and are increasingly used for LVT, their efficacy and safety in this setting remain uncertain due to limited and contradictory evidence.

Regarding the treatment, the RED VELVT observational study is, to date, the largest study comparing NOACs to VKAs for the treatment of LVT. In this multicentre cohort study of anticoagulation strategies for LVT, NOAC treatment was associated with a higher risk of systemic embolism compared with VKA use, even after adjustment for other factors.^45^ These results challenge the previous assumption of equivalence of NOACs with VKAs for LVT and highlight the need for prospective RCTs to determine the most effective treatment strategies for LVT. However, the retrospective nature of this study also predisposes it to result bias. Subsequently, three small-scale RCTs found NOACs to be non-inferior to VKAs.^8^,^10^ Further meta-analyses confirmed the findings of the above RCTs.^46 47^ These studies highlight that NOACs versus VKAs perhaps provide at least equivalent benefit-risk profiles in clinical settings where LVT exists. Yet, owing to the conflicting results of the RED VELVT and current RCTs,^45^ no consensus on the issue was formed. Regarding prevention, RCTs specifically focusing on the use of NOACs for primary prevention in patients at high risk for LVT are rare. Most RCTs have been conducted to evaluate the optimal antithrombotic regimen for the prevention of major adverse events in other similar clinical settings, such as heart failure or acute coronary syndromes. Similarly, the results vary. Results from the COMMANDER HF Trial indicate that very low dose rivaroxaban was not associated with a significantly lower rate of death, myocardial infarction or stroke than placebo among patients with worsening chronic heart failure, reduced LVEF, coronary artery disease and no AF.^48^ Conversely, results from the ATLAS ACS 2-TIMI 51 Trial indicate that in patients with a recent acute coronary syndrome, rivaroxaban reduced the risk of the composite endpoint of death from cardiovascular causes, myocardial infarction, or stroke and did not increase the risk of fatal bleeding.^49^ These varying results potentially indicate that differences in thrombosis in the various clinical settings may reasonably translate into differences in antithrombotic activity, resulting in different anticoagulant responsiveness. Recently, our team performed a modest-sized single-centre, open-label randomised trial of 279 patients and specifically examined whether low-dose anticoagulation (rivaroxaban 2.5 mg two times per day for 30 days) in addition to DAPT could decrease the risk of LV thrombus compared with DAPT alone.^7^ The addition of low-dose rivaroxaban compared with no such therapy lowered the risk of LV thrombus formation, as well as net adverse clinical events, without increased bleeding. This strategy on NOACs seems promising for use in such selected patients, but the limitations (eg, single-centre, isolated, open-label) of the trial also affected the generalisability. We summarise the still ongoing RCTs of NOACs for the treatment and prevention of LVT (table 3), and expect that future publication of these data will provide new insights for this area.

**Table 3:** Ongoing RCTs of new OACs for the treatment and prevention of LVT

Trial	Country	Year	Sample size	Design	Primary endpoint
NCT06013020	China	1 April 2024 to 31 December 2027	374	Experimental: The first month: rivaroxaban 2.5 mg two times per day plus standard DAPT. The following 11 months: lower-dose ticagrelor 60 mg two times per day (45 mg two times per day if <50 kg, ≥75 years) or clopidogrel (75 mg daily) plus aspirin (100 mg daily). Control: The first month: standard DAPT. The following 11 months: lower-dose ticagrelor or clopidogrel plus aspirin.	Efficacy endpoint: The incidence of LVT formation. Safety endpoint: The incidence of clinically significant bleeding according to ISTH criteria.
NCT05028777	Switzerland	1 January 2021 to 28 February 2026	550	All patients diagnosed with LVT through different imaging modalities (echocardiography, CT or CMR)	Primary combined endpoint includes the occurrence at 1 year of bleeding BARC type 2, 3 or 5.
NCT05973188	Pakistan	1 May 2023 to 30 June 2024	141	Group A: Warfarin group with dose-adjusted, target INR 2–3. Group B: Rivaroxaban 20 mg once a day. Group C: Apixaban 5 mg two times per day or 2.5 mg two times per day.	Presence or absence of LVT at 1 month, 3 months and 6 months follow-up on echocardiogram.
NCT05794399	Nepal	19 June 2023 to April 2025	196	Experimental: the patients with postmyocardial infarction and LVT will be treated with rivaroxaban 20 mg or 15 mg as indicated. Control: the patients with postmyocardial infarction and LVT will be treated with warfarin with a dose adjusted with the INR range of 2.0 to 3.0.	The resolution rate of LVT as assessed by cardiac CMR at the end of 3 months of the study period.
NCT04970576	Pakistan	25 June 2021 to 30 January 2024	320	Intervention group: Rivaroxaban 20 mg once a day for 3 months. Control group: warfarin therapy dose adjusted as per the target INR of 2 to 3.	Evaluate the efficacy of rivaroxaban in resolution of post-MI LVT as compared with standard warfarin therapy at the interval of 1 month and 3 months.
NCT03764241	China	1 February 2020 to 20 December 2023	280	DAPT + rivaroxaban group: Rivaroxaban 15 mg once a day will be used for 3 months unless there is a serious safety result. DAPT + warfarin group: Warfarin (INR 2.0–2.5) will be used for 3 months unless there are serious security consequences.	The LVT resolved will be determined monthly by follow-up imaging examination (CMR or TTE). The percentage of LVT resolved at 3 months will be calculated for each group.
NCT05892042	China	1 May 2023 to 1 April 2025	320	Experimental: intervention: the patient will receive rivaroxaban 15 mg daily in addition to the dual antiplatelet therapy. Control: patient will receive dual antiplatelet therapy.	The percentage of participants with first occurrence of stroke and other systemic embolism was evaluated at 1 year.

BARC, Bleeding Academic Research Consortium; CMR, cardiovascular MR; DAPT, dual antiplatelet therapy; INR, international normalised ratio; ISTH, International Society on Thrombosis and Hemostasis; LVT, left ventricular thrombus; MI, myocardial infarction; OAC, oral anticoagulant; RCT, randomised clinical trial; TTE, transthoracic echocardiography.

### Future

A critical unmet need in the management of LVT is the development of a systematic, stratified approach to predicting individual patient risks. Previous studies have identified several potential predisposing factors, including ventricular aneurysm, low LVEF, and alterations in coagulation and inflammatory markers. The relative weight of these factors in influencing LVT risk remains poorly understood. Quantifying and integrating LVT predisposing factors to stratify individual risk can significantly enhance the effectiveness of preventative interventions aimed at preventing life-threatening complications. Despite its clinical importance, there is currently a lack of established guidelines for LVT risk stratification. While Weinsaft and Holzknecht have proposed two simple risk prediction scores, their limited sample sizes necessitate further validation before widespread clinical adoption.^26 50^

The role of prophylactic anticoagulation in preventing LVT remains a subject of ongoing debate. While major guidelines, including the 2012 American College of Chest Physicians, 2013 American College of Cardiology/American Heart Association (AHA) STEMI, and 2014 American Stroke Association/AHA stroke guidelines^6 41 51^ have historically recommended VKA anticoagulation for patients at high risk of LVT, the 2017 European Society of Cardiology guidelines on STEMI notably completely fail to mention this recommendation,^4^ potentially reflecting evolving perspectives on limitations of VKA. Over the past 5 years, diagnostic and treatment protocols for LVT have largely overlooked the potential benefits of prophylactic anticoagulation. Despite limited research, the incidence of MACE in patients following LVT remains substantial, reaching up to 37.1%.^1^ This underscores the urgent need for RCTs to evaluate the efficacy of prophylactic anticoagulation in this population.

Finally, there is a lack of high-quality evidence to guide optimal therapy selection for patients with LVT. Additionally, the appropriate dosage of NOACs for patients with LVT, especially those receiving DAPT, remains unclear. The lack of definitive evidence poses a challenge for clinicians when selecting an antithrombotic regimen for LVT. Careful consideration of the risks and benefits is crucial.

## Conclusions

LVT remains a challenging complication, with many aspects of its management still existing in a grey area of evidence. While echocardiography continues to be the primary diagnostic tool, the use of CMR or other CT scans should be considered in cases of ambiguous echocardiographic results or when selecting high-risk patients, as these modalities can enhance diagnostic accuracy. To reduce the morbidity and mortality associated with LVT, it is essential to implement optimal anticoagulation strategies after carefully weighing the risks of bleeding. Due to the limited evidence and the lack of information regarding the optimal duration and dosing of treatment, future studies should address these remaining issues to improve patient outcomes.
